# Machine learning algorithm accurately detects fMRI signature of vulnerability to major depression

**DOI:** 10.1016/j.pscychresns.2015.07.001

**Published:** 2015-08-30

**Authors:** João R. Sato, Jorge Moll, Sophie Green, John F.W. Deakin, Carlos E. Thomaz, Roland Zahn

**Affiliations:** a Center for Mathematics, Computation, and Cognition, Universidade Federal do ABC, Bangu, Santo André 09020-040, Brazil; b Cognitive and Behavioral Neuroscience Unit and Neuroinformatics Workgroup, D'Or Institute for Research and Education (IDOR), Rio de Janeiro 22281-100, Brazil; c The University of Manchester & Manchester Academic Health Sciences Centre, School of Psychological Sciences, Neuroscience and Aphasia Research Unit, Manchester M13 9PL, UK; d The University of Manchester & Manchester Academic Health Sciences Centre, Institute of Brain, Behaviour and Mental Health, Neuroscience & Psychiatry Unit, Manchester M13 9PL, UK; e Department of Electrical Engineering, Centro Universitario da FEI, Sao Bernardo do Campo 3972, Brazil; f Institute of Psychiatry, Psychology, and Neuroscience, King's College London, Department of Psychological Medicine, Centre for Affective Disorders, London SE5 8AZ, UK

**Keywords:** Self-blame, Major depressive disorder, Anterior temporal lobe

## Abstract

Standard functional magnetic resonance imaging (fMRI) analyses cannot assess the potential of a neuroimaging signature as a biomarker to predict individual vulnerability to major depression (MD). Here, we use machine learning for the first time to address this question. Using a recently identified neural signature of guilt-selective functional disconnection, the classification algorithm was able to distinguish remitted MD from control participants with 78.3% accuracy. This demonstrates the high potential of our fMRI signature as a biomarker of MD vulnerability.

## Highlights

• We use machine learning to test a new fMRI biomarker of depression vulnerability.
• This is based on guilt-selective anterior temporal functional connectivity changes.
• The classification algorithm uses Maximum Entropy Linear Discriminant Analysis.
• The remitted depression group is distinguished from controls with 78.3% accuracy.
• This shows a high biomarker potential for detecting vulnerability to depression.

## 1 Introduction

Individuals who have experienced at least one major depressive (MD) episode are at a three to six times greater risk of developing future MD episodes than those with no history of MD (Eaton et al., 2008). Therefore, investigations of remitted MD have the potential to identify imaging biomarkers of vulnerability to MD (Bhagwagar and Cowen, 2008). The identification of such biomarkers is crucial for neurobiological and experimental medicine studies.

Using a standard statistical analysis of functional magnetic resonance imaging (fMRI) data, we have previously demonstrated that patients with remitted MD exhibited a functional disconnection between the anterior temporal lobe (ATL) and frontal-subcortical regions, when compared with a control group with no history of MD (Green et al., 2012). We further demonstrated that this ATL functional disconnection selectively occurred when patients experienced guilt relative to indignation towards others during the fMRI scan. This neural signature accounted for the well-known tendency of overgeneralizing self-blame and guilt in MD (Green et al., 2013). This group-level standard analysis, however, is unable to answer the clinical question of whether this particular fMRI signature has the potential to serve as a biomarker to detect vulnerability in the individual. Machine learning algorithms have been successfully used to assess the potential of fMRI signatures to serve as biomarkers of current MD (Fu et al., 2008, Marquand et al., 2008, Zeng et al., 2014). These algorithms attain powerful discriminative ability by being trained on a dataset to arrive at an optimal model that separates two classes of data (Sato et al., 2008, Lee et al., 2010, Deshpande et al., 2013). This is achieved through the ability to condense multiple variables (e.g., MRI signals in each voxel of the brain) into a measure that captures the complex multivariate patterns arising from these variables. Machine learning algorithms thereby also capture interactions between brain regions highlighted to be of importance in network models of MD (Seminowicz et al., 2004). To our knowledge, there has been no study that has applied this approach to patients with remitted MD in order to identify potential fMRI biomarkers of MD vulnerability. Here, we used a particular machine learning algorithm, Maximum Entropy Linear Discriminant Analysis (MLDA) (Sato et al., 2008, 2011), to address this question.

## 2 Methods

Participants provided written informed consent as approved by the South Manchester NHS Research Ethics Committee. Participants in the MD disorder group (*n*=25, 16 medication-free) fulfilled criteria for a past major depressive episode according to the Diagnostic and Statistical Manual IV-TR (American-Psychiatric-Association, 2000) and were in remission for at least 12 months. Exclusion criteria were current axis-I disorders and a history of alcohol or substance abuse or past co-morbid axis-I disorders being the likely primary cause of the depressive syndrome; see Green et al. (2012) for further clinical details. The healthy control group (*n*=21) had no current or past axis-I disorders and no first degree family history of MD, bipolar disorder, or schizophrenia. Standard fMRI analyses on the same participants have been previously reported (Green et al., 2012). The groups were matched on age, gender and years of education (Green et al., 2012).

### 2.1 fMRI paradigm

Participants saw written statements describing actions counter to social and moral values described by social concepts (e.g., “stingy”, “boastful”) in which the agent was either the participant (“self-agency” condition, *n*=90) or their best friend (“other-agency” condition, *n*=90). Self- and other-agency conditions used the same social concepts (self-agency: e.g., “[participant's name] does act stingily towards [best friend's name]”; other-agency: e.g., “[best friend's name] does act stingily towards [participant's name]”). In addition, we used a low-level resting-state baseline condition: fixation of visual pattern with no button press (*n*=90). Stimuli were presented in an event-related design for a maximum of 5 s within which participants had to make a decision whether they would feel “extremely unpleasant” or “mildly unpleasant” from their own perspective.

After the scanning session, participants rated the unpleasantness of each action (7-step scale visual analog Likert scale) in order to control for the degree of negative valence and emotional intensity. Furthermore, participants were required to “choose one feeling that (they) would feel most strongly” from the following list: guilt, contempt/disgust towards self, shame, indignation/anger towards self, indignation/anger towards others, contempt/disgust towards others, none, other feeling. “Guilt” and “indignation towards others” trials for the fMRI analysis were defined by individual post-scanning ratings and there were no differences in unpleasantness or frequency of these two emotions between groups (Green et al., 2012).

### 2.2 Image acquisition

Four hundred gradient-echo planar images with T2*-weighted volumes were acquired (in each of the 3 runs) on an MR system (3-T Achieva, Philips) equipped with an eight-channel coil (axial slice thickness=3 mm; ascending; repetition time=2000 ms; echo time=20.5 ms; field of view=220×220×120 mm^3^; matrix size=80×80, 2.29×2.29×3 mm^3^). The sequence had been optimized for measuring ventral brain regions (Green et al., 2012).

### 2.3 Analysis

The images were processed in Statistical Parametric Mapping Version 8 (SPM8) (www.fil.ion.ucl.ac.uk/spm) by head realignment, unwarping, spatial normalization (Montreal Neurological Institute-152 template) and smoothing (full width at half-maximum=6 mm). Psychophysiological interaction (PPI) analysis (Friston et al., 1997) was carried out using the right superior anterior temporal lobe (ATL) as the seed region of interest (Green et al., 2012), spherical, radius=4 mm at *x*=58, *y*=0, *z*=−12). Finally, PPI *t*-maps of each individual for the guilt vs. indignation contrast at the first level were masked (gray-matter coefficient >0.25, (Green et al., 2012)) and considered as input to the classifier. To control for the specificity of classification, we ran a separate classification analysis using the *t*-maps for the effects of the ATL seed signal irrespective of psychological condition. The classification was carried out by using MLDA with feature selection for the 1% most discriminative voxels with a maximum entropy criterion, but no parameter setting (e.g., cost) (Sato et al., 2011). The accuracy of the classifier in distinguishing the groups based on participants’ PPI maps was calculated by using the standard leave-one-subject-out procedure in which the classification is cross-validated iteratively by using a model based on the sample after excluding one subject to independently predict group membership.

## 3 Results

**Fig. 1:**
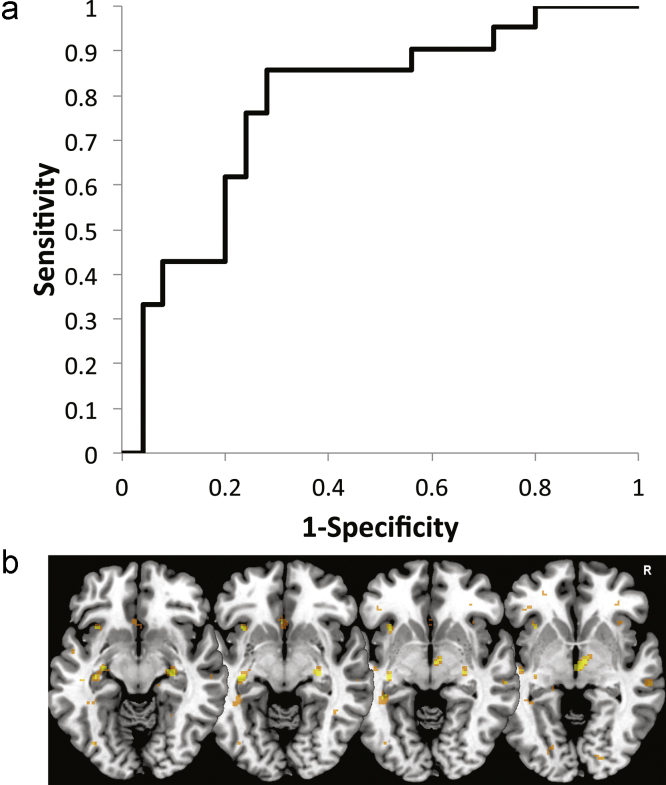
(a) Receiver Operator Characteristic (ROC) curve for the MLDA classifier's ability to distinguish MD from control images, based on the projected values (decision values). (b) Axial slices ventral to the corpus callosum display MLDA weight vector maps highlighting the voxels which were among the 1% most discriminative for MD patients vs. controls including the subgenual cingulate cortex, both hippocampi, the right thalamus and the anterior insulae.

The MLDA algorithm was able to distinguish MD from control participants with an accuracy of 78.26% when using the leave-one-out method of cross-validating the classifier (*p*<0.001, binomial test, area under the curve (AUC)=0.781, *p*=0.001, chi-square test). This corresponded to a sensitivity of 72.00% with a specificity of 85.71% (see Fig. 1). Montgomery–Åsberg Depression Scale scores (Montgomery and Åsberg, 1979), as standard observer-rated measures of depression severity, were not correlated with the decision values (*r*=−0.05, *p*=0.70), which suggests that the classifier captured correlates of trait vulnerability factors rather than state markers of residual depressive symptoms. Finally, there was no difference in the classifier decision values between MD patients currently taking antidepressants and those who were medication-free (Mann–Whitney test, *W*=49, *p*=0.21).

To probe the specificity of the chosen biomarker, we carried out a control classification analysis using functional connectivity maps of the ATL seed region irrespective of psychological condition. The MLDA algorithm was unable to accurately distinguish MD from control groups using this information (accuracy=39%).

## 4 Discussion

Our results demonstrate that guilt-selective changes in functional connectivity of the ATL are sufficient to distinguish the remitted MD group from the control group with high accuracy. The supporting analyses showed that this distinction is not based on residual depressive symptoms or effects of antidepressant medication. In addition, our control analysis demonstrated that functional connectivity of the ATL irrespective of the psychological experience (guilt or indignation), is not distinctive of MD. This highlights the relevance of self-blaming emotions and their neural correlates to the pathophysiology of MD (Green et al., 2012, Jankowski and Takahashi, 2014).

On a cautionary note, the difference in vulnerability to MD between our study groups cannot be further characterized into primary MD vulnerability (i.e., before the first episode) and secondary vulnerability as a result of scar effects of previous episodes. Further, because the sample comprised fully remitted MD patients with no relevant co-morbidity, this study may not generalize to chronic MD with co-morbid conditions. Future studies are needed to test the reproducibility of the classifier in an independent sample and might use optimized feature selection to improve stability.

Taken together, these findings show that guilt-selective functional disconnection of the ATL has the potential to be further developed into a clinically useful fMRI biomarker of MD vulnerability. To achieve this, a further validation study is currently being carried out to test whether guilt-selective functional disconnection is able to prospectively predict recurrence of MD over the next year in patients who have stopped their antidepressant treatment.

## Financial disclosures

None.
